# Plasma Proteomics for Predicting Cardiovascular Disease: How Many Proteins are Enough?

**DOI:** 10.1007/s12265-025-10713-z

**Published:** 2025-10-10

**Authors:** Patrick Royer, Elias Björnson, Martin Adiels, Anders Gummesson, Göran Bergström

**Affiliations:** 1 Department of Molecular and Clinical Medicine, Sahlgrenska Academy, Institute of Medicine, Gothenburg University, Gothenburg, Sweden; 2 Department of Clinical Physiology, Region Västra Götaland, Sahlgrenska University Hospital, Gothenburg, Sweden; 3 Department of Critical Care, University Hospital of Martinique, Fort-de-France, Martinique, French West Indies France; 4 School of Public Health and Community Medicine, Institute of Medicine, University of Gothenburg, Gothenburg, Sweden; 5 Department of Clinical Genetics and Genomics, Region Västra Götaland, Sahlgrenska University Hospital, Gothenburg, Sweden

**Keywords:** Proteomics, Cardiovascular diseases, Risk factors, Machine learning

High-throughput proteomics technologies have enabled the measurement of an increasing number of plasma proteins. To date, the antibody-based Olink Explore HT platform can quantify up to 5,400 proteins, while the aptamer-based Somascan platform can measure more than 11,000 proteins. Applying machine learning methods to these large-scale proteomics datasets allows for the refinement of existing clinical models for predicting cardiovascular disease [1]. However, the number of proteins included in the different published predictive models varies substantially, raising questions about the impact of protein quantity on predictive capacity [1]. The aim of our exploratory study was to investigate how the number of proteins affects the performance of models trained to predict major cardiovascular events (MACE) in primary prevention settings.

The study population consisted of participants from the UK Biobank, who were randomly selected for plasma proteomic analysis using Olink Explore 3072 during their baseline visit [2]. Participants with a prior history of MACE or with more than 20% missing protein data were excluded. MACE was defined as fatal or non-fatal myocardial infarction, ischaemic stroke, and intracerebral haemorrhage during the 10-year follow-up.

Details on proteomics technology and data handling have been outlined previously [3]. Briefly, 2,941 plasma protein analytes were quantified using the Olink Explore 3072 platform. Proteins with more than 20% missing values were excluded, and other missing data were imputed using the K-nearest neighbors algorithm.

The full dataset was randomly divided into a training set (80%) for development and a test set (20%) for validation. Extreme gradient boosting (XGB) machine learning models [4], including an increasing number of proteins, were trained to predict the 10-year risk of MACE. The XGB algorithm builds an ensemble of shallow trees sequentially, with each tree correcting the errors of the previous ones. This approach, combined with built-in regularization techniques, helps reduce overfitting and improve model generalization. Early stopping and fivefold cross-validation were implemented to further prevent overfitting. Two types of models for protein selection were used: models with randomly selected proteins and models with data-driven protein selection using the Minimum Redundancy Maximal Relevance feature selection algorithm based on mutual information [5]. The protein sets used to train the models were incrementally increased, starting from one up to 500, and then by 10 up to 2,919. This sequence resulted in 742 protein sets for both random and data-driven models. The sequence was repeated 10 times using bootstrapped versions of the training set and different random seeds. The performance of the models was assessed using the area under the receiver operating characteristic curve (AUC). For each of the 742 protein steps, the mean AUC was computed for the 10 random protein models and the 10 data-driven protein models (Fig. 1a).

The protein models were compared to a clinical risk model trained using 10 traditional risk factors: age, sex, systolic blood pressure, current smoking status, diabetes status and age at diagnosis, glycated haemoglobin, estimated glomerular filtration rate, high-density lipoprotein, and total cholesterol.

Machine learning analyses were performed using R version 4.0.4 (R Foundation for Statistical Computing, Vienna, Austria).

A total of 2,919 proteins were measured in 38,273 participants without a previous history of MACE. Among them, 1,364 individuals experienced MACE, representing 3.6% of the cohort. Approximately 100 proteins, selected using the data-driven technique, resulted in a model with similar performance to the clinical risk model (AUC = 0.72). In contrast, around 1,000 randomly selected proteins were needed to achieve equal performance. There was a gradual improvement in the performance of both the data-driven and the random models as more proteins were added, with performance not peaking until all proteins were included (max AUC = 0.73) (Fig. 1b).

These results highlight the importance of carefully selecting proteins to achieve high prediction performance at a minimal cost. Furthermore, within the limits of our targeted protein panel, the prediction performance appeared to continuously increase as more proteins were added. This suggests that deeper phenotyping could enhance prediction in ways that traditional clinical factors cannot match. However, the technical complexity and high cost of comprehensive plasma proteomics remain significant obstacles to clinical implementation.

## Clinical Relevance

The continuous improvement in predictive performance with increasing protein count suggests that comprehensive plasma proteomics could provide clinically valuable insights beyond traditional risk factors, although current costs and complexity limit routine implementation.

**Fig. 1:**
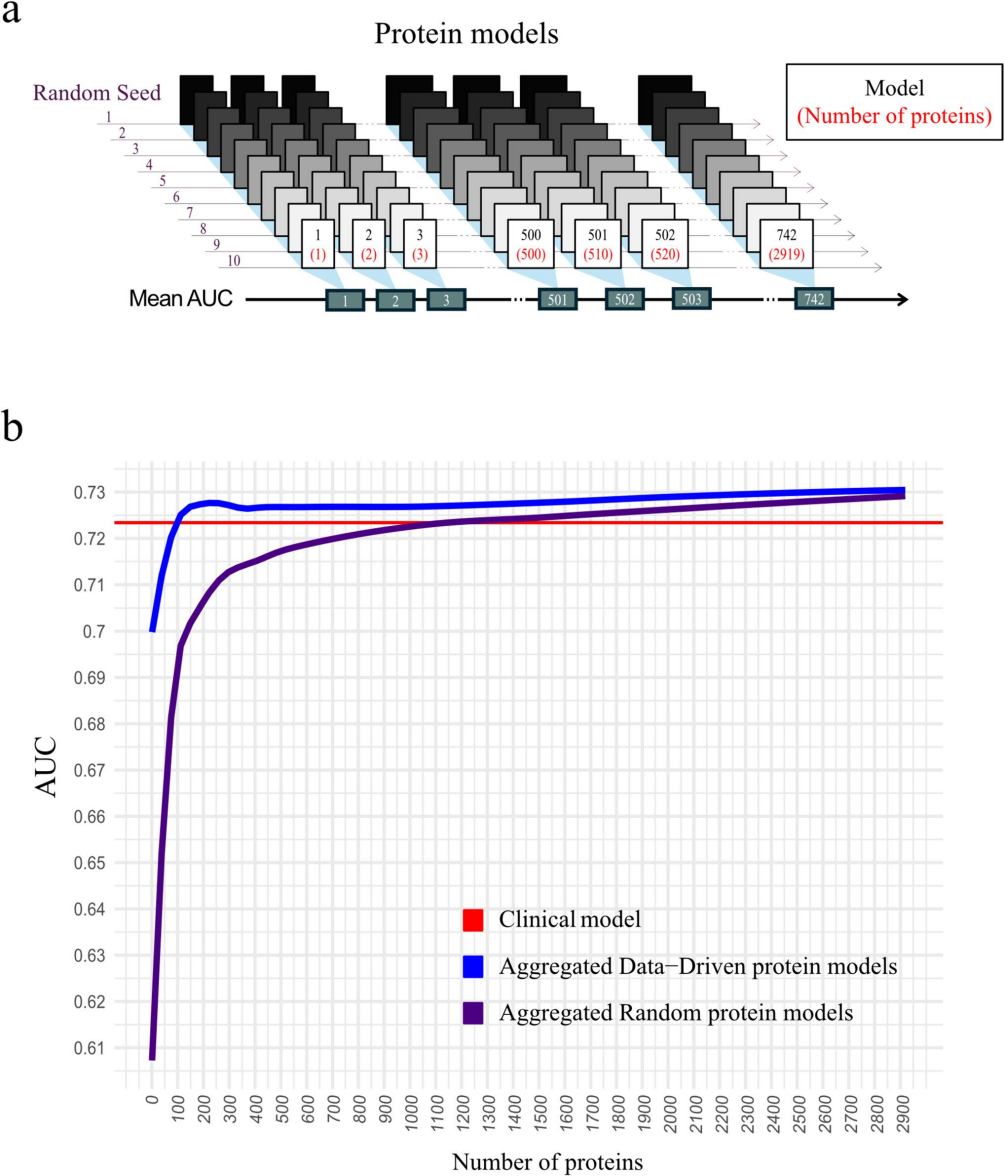
**a** Construction of the random and data-driven protein models using the same process. **b** Performance of the random and data-driven protein models compared to the clinical model as the number of proteins increases

## Data Availability

This research was conducted using data from the UK Biobank (Application Number 82018). The data used in this study are not publicly available due to data access restrictions, but are available to bona fide researchers upon application to the UK Biobank: https://www.ukbiobank.ac.uk/enable-your-research/apply-for-access
